# Draft Genome Sequence of *Lactobacillus rhamnosus* NRRL B-442, a Potential Probiotic Strain

**DOI:** 10.1128/genomeA.00046-18

**Published:** 2018-02-15

**Authors:** Muhammed Shafeekh Muyyarikkandy, Fahad H. Alqahtani, Ion Mandoiu, Mary Anne Amalaradjou

**Affiliations:** a Department of Animal Science, University of Connecticut, Storrs, Connecticut, USA; b Computer Science and Engineering Department, University of Connecticut, Storrs, Connecticut, USA

## Abstract

Lactic acid bacteria are known to exhibit probiotic properties through various mechanisms, including competitive exclusion, pathogen inhibition, production of antimicrobial substances, and maintenance of eubiosis. Here, we present the draft genome sequence of a novel probiotic strain, *Lactobacillus rhamnosus* strain NRRL B-442, which exhibits potent antivirulence activity against *Salmonella enterica*.

## GENOME ANNOUNCEMENT

Probiotics, including members of the genus *Lactobacillus*, play an important role in maintaining host health by maintaining microbial balance, immunomodulation, and protection against infections (1). Besides their effects on the host, these microorganisms also exert a direct effect on the invading pathogens. This antimicrobial effect is mediated by the production of antimicrobial substances, competitive exclusion, antitoxin effect, antiadhesive and anti-invasive effects, and attenuation of virulence (1–3). In this regard, recent studies conducted using *in vitro* models demonstrated the ability of *Lactobacillus rhamnosus* strain NRRL B-442 via the modulation of *Salmonella* genes critical for host colonization to inhibit motility, adhesion and invasion in primary cecal epithelial cells, and invasion and survival in chicken macrophages for *Salmonella enterica *serovar Enteritidis, *Salmonella enterica* serovar Heidelberg, and *Salmonella enterica* serovar Typhimurium DT 104 (4).

*L. rhamnosus* NRRL B-442 was grown in de Man-Rogosa-Sharpe broth at 37°C for 24 h prior to DNA extraction. A paired-end library was created using the MiSeq platform (Illumina) in the Microbial Analysis, Resources, and Services Facility at the University of Connecticut (Storrs, CT) with an average insert size of 550 and an average read length of 251 bp. Quality checks were performed using FastQC v0.11.5 (http://www.bioinformatics.babraham.ac.uk/projects/fastqc/). Adaptors, primers, and bases with a Phred score of <20 were trimmed using Trimmomatic v3.10.1 (5) with a headcrop of 15. The SPAdes genome assembler v.3.10.1 (6) was used for the *de novo* assembly of paired-end reads to create 111 contigs, and any contigs with less than 200 bp were discarded. Genome annotations were carried out using the Rapid Annotations using Subsystems Technology (RAST) server (7) and the NCBI Prokaryotic Genome Automatic Annotation Pipeline (PGAAP) (8).

The 2,974,909-bp genome is composed of a single circular chromosome with a G+C content of 46.7%. The chromosome contains 2,989 coding sequences and 75 RNA genes as predicted by PGAAP (14 rRNA, 58 tRNA, and 3 noncoding RNA [ncRNA] genes). There are 333 subsystems represented in the chromosome. According to the RAST analysis, 1,826 protein-coding genes were assigned to putative functional categories, with the most abundant being related to carbohydrate metabolism (27%) and protein metabolism (11%). Furthermore, functional analysis revealed that the majority of the genes involved in carbohydrate metabolism were associated with disaccharide uptake and synthesis. Additionally, the presence of multiple genes involved in bacteriocin and colicin synthesis were detected. Similar to other strains of the species, *L. rhamnosus* NRRL B-442 was found to contain genes responsible for exopolysaccharide biosynthesis. No remarkable virulence-associated genes were found. The genome information presented here will help further specific studies of this strain and to exploit its probiotic potential.

### Accession number(s).

This whole-genome shotgun project has been deposited at DDBJ/ENA/GenBank under the accession number PKQF00000000. The version described in this paper is version PKQF01000000.
